# Rapid and Visual Detection of Type 2 Porcine Reproductive and Respiratory Syndrome Virus by Real-Time Fluorescence-Based Reverse Transcription Recombinase-Aided Amplification

**DOI:** 10.3390/v14112526

**Published:** 2022-11-15

**Authors:** Wenlong Xia, Yao Chen, Xue Ding, Xiaoming Liu, Huipeng Lu, Changming Guo, Hua Zhang, Zhijun Wu, Jing Huang, Zhongjun Fan, Shupei Yu, Huaichang Sun, Shanyuan Zhu, Zhi Wu

**Affiliations:** 1 Jiangsu Key Laboratory for Bioresources of Saline Soils, School of Ocean and Biological Engineering, Yancheng Teachers University, Yancheng 224007, China; 2 College of Veterinary Medicine, Jiangsu Co-Innovation Center for Prevention and Control of Important Animal Diseases and Zoonoses, Yangzhou University, Yangzhou 225009, China; 3 Jiangsu Key Laboratory for High-Tech Research and Development of Veterinary Biopharmaceuticals, Jiangsu Agri-Animal Husbandry Vocational College, Taizhou 225300, China; 4 School of Pharmacy, Yancheng Teachers University, Yancheng 224007, China; 5 Jiangsu Province Engineering Research Center of Tumor Targeted Nano Diagnostic and Therapeutic Materials, Yancheng Teachers University, Yancheng 224007, China; 6 Yancheng Animal Husbandry and Veterinary Station, Yancheng 224001, China

**Keywords:** porcine reproductive and respiratory syndrome virus, real-time fluorescence-based reverse transcription recombinase-aided amplification, rapid detection

## Abstract

Porcine reproductive and respiratory syndrome (PRRS) is one of the most important diseases that has brought significant economic losses to the swine industry worldwide. Rapid and accurate PRRS virus (PRRSV) detection is one of the key factors for PRRS prevention and control. This study developed a real-time fluorescence-based reverse transcription recombinase-aided amplification (RF-RT-RAA) method for type 2 PRRSV (PRRSV-2) detection. The RF-RT-RAA assay could be performed at 42 °C for 20 min with the optimal primers and a probe. RF-RT-RAA results could be monitored using real-time fluorescence read-out or visually observed with the naked eye using a portable blue light transilluminator. The method had a strong specificity; no cross-reaction was identified with the detected common swine viruses. Moreover, the technique yielded high sensitivity with the lowest detection limit of 10^1^ copies/μL and exhibited good repeatability and reproductively with the coefficients of variation (CV) less than 10%. Eighty-seven clinical samples were tested using RF-RT-RAA and a commercial PRRSV-2 RT-qPCR detection kit. The coincidence rate was 100% between RF-RT-RAA (real-time fluorescence read-out) and RT-qPCR, and 97.7% between RF-RT-RAA (visually observed) and RT-qPCR. The RF-RT-RAA assay provides a new method for rapid and visual detection of PRRSV-2.

## 1. Introduction

Porcine reproductive and respiratory syndrome virus (PRRSV) is an enveloped, positive-stranded RNA virus belonging to the *Arteriviridae* family and *Nidovirales* order [1]. It is the causative agent of porcine reproductive and respiratory syndrome (PRRS), a severe infectious disease that causes reproductive failures in sows and respiratory problems in pigs of all ages [2]. Since its discovery in North America in 1987, PRRSV has spread worldwide, causing immense economic losses in the global swine industry. Based on the genomic and antigenic diversity, PRRSVs are divided into two genotypes, the European genotype (PRRSV-1) and the North American genotype (PRRSV-2), which differ by approximately 40% at the genomic level [3,4]. In China, PRRSV was first isolated and identified as PRRSV-2 in 1996 and has been endemic ever since [5]. Currently, most described PRRSV isolates from China belong to PRRSV-2, including classical PRRSV (C-PRRSV), highly pathogenic PRRSV (HP-PRRSV), NADC30-like PRRSV, and NADC34-like PRRSV [6,7,8]. The existing vaccines lack cross-immune protection due to the high diversity of PRRSV strains, and no effective commercial medicines are available for controlling PRRSV infection [9]. Consequently, early diagnosis is critical for PRRS prevention and control. Rapid and accurate PRRSV detection is the prerequisite for PRRS diagnosis.

The current methods for detecting PRRSV mainly include virus isolation, indirect immunofluorescence assay (IFA), enzyme-linked immunosorbent assay (ELISA), reverse transcription polymerase chain reaction (RT-PCR), and quantitative RT-PCR (RT-qPCR) [10,11,12]. Virus isolation and IFA require time-consuming cell cultures. Moreover, many field PRRSV strains have lost their ability to infect Marc-145 cells, the most extensively used cells for PRRSV isolation [13]. ELISA requires specific antibodies and complicated procedures, and it is not suitable for early diagnosis [14]. Due to their high sensitivity and specificity, RT-PCR and RT-qPCR have been widely used for PRRSV detection in many laboratories. However, both depend on sophisticated thermal cycling instruments and skilled technicians, which limits their application in basic laboratories or fields. Accordingly, developing a fast, simple, and accurate on-site diagnostic assay for PRRSV detection remains necessary.

**Figure 1:**
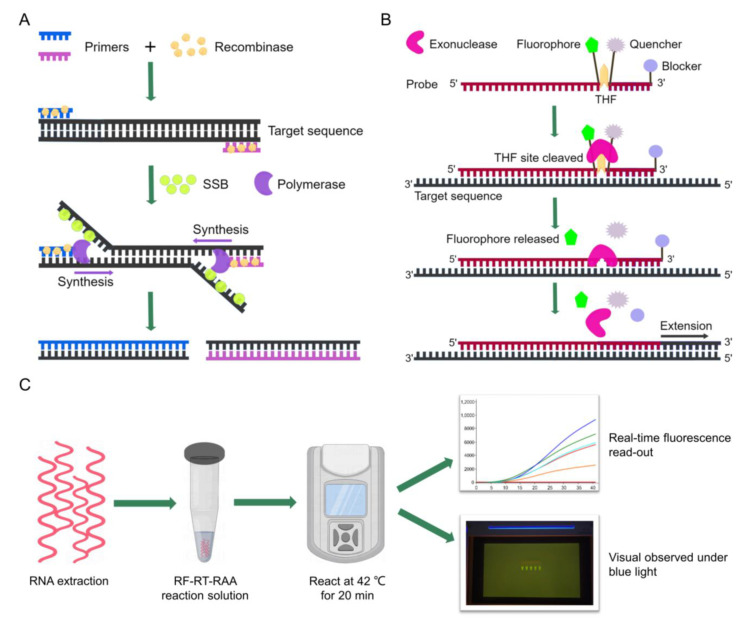
Schematics of RAA-based assay mechanism and RF-RT-RAA detection workflow. (**A**) Process of RAA reaction. (**B**) Principle of real-time fluorescence RAA. (**C**) Workflow of RF-RT-RAA detection. SSB, single-strand DNA binding protein; THF, tetrahydrofuran; RF-RT-RAA, real-time fluorescence-based RT-RAA. This figure was drawn by Figdraw online software (https://www.figdraw.com/ (accessed on 15 September 2022)).

Recombinase-aided amplification (RAA) is a novel isothermal nucleic acid amplification technique that does not require complex thermal cycles and can be operated at a constant temperature (37–42 °C) in a short time (15–30 min) [15]. The RAA reaction relies on three essential proteins: a recombinase, a single-strand DNA binding protein (SSB), and a DNA polymerase. In RAA, the recombinase pairs specific primers to the homologous sequence in DNA template, then a chain exchange reaction occurs and SSB helps to open the double-stranded structure of DNA. Subsequently, DNA polymerase initiates the target region amplification [16] (Figure 1A). Reverse transcription RAA (RT-RAA) can amplify RNA template in a one-step method, saving time and cost. Additionally, RAA/RT-RAA results could be analyzed via real-time fluorescence if a special probe was added to the reaction mixture. The probe contained a tetrahydrofuran (THF) site, with a fluorophore and a quencher labeled on both sides, and a blocker was added to the 3′ end of the probe. The fluorophore was stable in the single-strand probe and its fluorescence was absorbed by the quencher when exonuclease was not activated. Once the probe bound to target sequence, exonuclease was activated, recognizing and cleaving the THF site. Then, the fluorophore was released and the fluorescence signal was able to be detected [17,18] (Figure 1B). Recently, real-time fluorescence-based RAA/RT-RAA has been successfully used for rapid detection of several viruses, such as severe acute respiratory syndrome coronavirus 2 (SARS-CoV-2) [19], duck circovirus [18], porcine epidemic diarrhea virus (PEDV) [20], and classical swine fever virus (CSFV) [21] due to its rapid, simple, and promising nature.

In this study, we developed a real-time fluorescence-based RT-RAA (RF-RT-RAA) assay for detecting PRRSV-2. RF-RT-RAA amplicons can be detected in real time with a portable fluorescence detector or be visualized at the endpoint by a portable blue light transilluminator (Figure 1C), which provides a new method for rapid and visual detection of PRRSV. In addition, its detection performance in clinical samples was evaluated by comparing with a commercial RT-qPCR kit.

## 2. Materials and Methods

### 2.1. Virus and Clinical Samples

PRRSV-2 strains JXA1, VR-2332, JS07, and SH1705 were provided by Jiangsu Key Laboratory for High-Tech Research and Development of Veterinary Biopharmaceuticals. The porcine pseudorabies virus (PRV) strain Bartha-K61, porcine epidemic diarrhea virus (PEDV) strain AJ1102, porcine circovirus type 2 (PCV2) strain DBN-SX07, transmissible gastroenteritis virus (TGEV) strain WH-1, and porcine parvovirus (PPV) strain S-1 were purchased from Huahong Biological Technology Co., Ltd., (Shandong, China). A total of 87 clinical samples were collected from lungs, lymph nodes, spleens, kidneys, and blood of dead or diseased pigs from different farms in Jiangsu Province of China. These pigs were initially diagnosed with PRRS with clinical symptoms of cough, dyspnea, fever, skin cyanosis, abortion, or loss of appetite. All the samples were stored in ice boxes after being collected, and transported to the laboratory immediately.

### 2.2. Nucleic Acid Extraction

PRRSV, PRV, PEDV, PCV2, TGEV, and PPV RNA/DNA templates were extracted using MiniBEST Viral RNA/DNA Extraction Kit (Takara Biotech Co., Ltd., Beijing, China). Clinical tissue samples (lungs, lymph nodes, spleens, and kidneys) were cut into small pieces (about 100 mg) using sterile scissors, and homogenized with a hand-held homogenizer (Lichen Instrument Technology Co., Ltd., Shanghai, China), then the total RNA was extracted using RNAiso Plus Reagent (Takara Biotech Co., Ltd., Beijing, China). Blood samples were collected for serum separation, and 200 µL of each serum sample was used for RNA extraction by RNAiso Blood Reagent (Takara Biotech Co., Ltd., Beijing, China).

### 2.3. Primer Screening and Probe Design

Typically, 50 ORF6 gene sequences of PRRSV-2 from China were downloaded from GenBank database; then, the sequences were aligned using the MegAlign program (Lasergene v7.0, DNASTAR, Inc., Madison, WI, USA). Three primer pairs (Table 1) were designed based on the conserved region of the ORF6 gene. All primers were confirmed for species specificity using the NCBI Nucleotide BLAST web tool (https://blast.ncbi.nlm.nih.gov/Blast.cgi (accessed on 10 January 2022)). RT-RAA reaction was performed using an RT-RAA (Basic) Kit provided by Weifang Amp-Future Biotech Co., Ltd. (Shandong, China), with the reaction system as follows: buffer A 29.4 μL, forward primer (10 mM) 2 μL, reverse primer (10 mM) 2 μL, ddH_2_O 12.1 μL, PRRSV (JXA1 strain) RNA template 2 μL, buffer B 2.5 μL. All components except template and buffer B were premixed and pipetted into the reaction tube containing dry enzyme powder of RT-RAA provided by the kit. Two microliters of RNA template and 2.5 μL buffer B were subsequently added to the mixture. RNA template was replaced for the negative control by 2 μL ddH_2_O. The reaction tube was incubated immediately in a water bath at 42 °C for 20 min. RT-RAA amplicon was purified using phenol–chloroform and analyzed by 2% agarose gel electrophoresis; then, the optimal primer pair was selected. The probe (Table 1) was designed based on the optimal primer pair. An in silico check for the optimal primer pair and the probe was performed using the NCBI Primer-BLAST web tool (https://www.ncbi.nlm.nih.gov/tools/primer-blast/ (accessed on 10 February 2022)), and no off target binding was determined. The primers and probe were synthesized by Sangon Biotech Co., Ltd. (Shanghai, China).

### 2.4. RF-RT-RAA Assay Establishment

According to the instructions of the RT-RAA (Fluorescence) Kit (Weifang Amp-Future Biotech Co., Ltd., Shandong, China), the reaction system was prepared as follows: buffer A 29.4 μL, forward primer (10 mM) 2 μL, reverse primer (10 mM) 2 μL, probe (10 mM) 0.6 μL, ddH_2_O 11.5 μL, PRRSV (JXA1 strain) RNA template 2 μL, buffer B 2.5 μL. All components except template and buffer B were premixed and pipetted into the reaction tube containing dry enzyme powder of RF-RT-RAA provided by the kit. Two microliters of RNA template and 2.5 μL buffer B were added to the mixture. The reaction tube was immediately placed in a WL-16-II portable thermostatic fluorescence detector (Weifang Amp-Future Biotech Co., Ltd., Shandong, China) and reacted for 20 min at 42 °C. The fluorescence signal was collected every 30 s. After the reaction, the products were visualized using a BL-20 portable blue light transilluminator (Labgic Biotech Co., Ltd., Beijing, China) at an exciting light wavelength of 470 nm.

### 2.5. Specificity Test

Under the abovementioned reaction system and conditions, RNA/DNA of PRRSV, PRV, PEDV, PCV2, TGEV, and PPV were used as templates for RF-RT-RAA assay, and ddH_2_O was used as a negative control instead of nucleic acid templates.

### 2.6. Standard Plasmid Construction

The RNA of PRRSV JXA1 was reverse transcribed to cDNA using a PrimeScript RT Master Mix (Takara Biotech Co., Ltd., Beijing, China). The cDNA was used as the template for PCR amplification of the PRRSV ORF6 gene with the primer pair ORF6 F1/ORF6 R1 (Table 1). PCR was conducted using a 2 × Rapid Taq Master Mix (Vazyme Biotech Co., Ltd., Nanjing, China) with the following reaction system: 2 × Rapid Taq Master Mix 25 μL, cDNA template 2 μL, forward primer (10 mM) 2 μL, reverse primer (10 mM) 2 μL, ddH_2_O 19 μL. PCR program was as follows: 95 °C for 3 min (predenaturation); 30 cycles of 95 °C for 15 s (denaturation), 58 °C for 15 s (annealing), and 72 °C for 15 s (extension); 72 °C for 5 min (final extension). The amplification product was purified using a FastPure Gel DNA Extraction Mini Kit (Vazyme Biotech Co., Ltd., Nanjing, China) and ligated to pUCm-T Vector (Beyotime Biotech Co., Ltd., Shanghai, China). The concentration of the resulting plasmid pUCm-ORF6 was measured, then the pUCm-ORF6 copy number was calculated as in a previous study [16].

### 2.7. RF-RT-RAA and RT-PCR Sensitivity Tests

Ten-fold dilutions of standard plasmid pUCm-ORF6 were prepared, ranging from 10^6^–10^0^ copies/μL, and used as templates for RF-RT-RAA and RT-PCR sensitivity tests. The RF-RT-RAA assay was conducted as mentioned above. RT-PCR assay was carried out with the same primers as RF-RT-RAA, and its reaction system was the same as those used in standard plasmid construction. The annealing temperature and extension time of PCR have been optimized, with the final program as follows: 95 °C for 3 min (predenaturation); 30 cycles of 95 °C for 15 s (denaturation), 56 °C for 15 s (annealing), and 72 °C for 15 s (extension); 72 °C for 5 min (final extension). In addtion, the PRRSV strain VR-2332 (10^4.5^ TCID_50_/mL) was 10-fold serial diluted, and viral RNA was extracted from each dilution using a MiniBEST Viral RNA/DNA Extraction Kit (Takara Biotech Co., Ltd., Beijing, China). These RNA samples were also used as templates for RF-RT-RAA sensitivity tests.

### 2.8. Repeatability and Reproducibility Tests

Three dilutions (10^3^, 10^2^, and 10^1^ copies/μL) of plasmid pUCm-ORF6 were used as templates for the RF-RT-RAA assay to test the intra-assay repeatability; each dilution reaction was repeated three times in one run. Each dilution was tested in three independent runs on separate days for inter-assay reproducibility. The coefficients of variation (CVs) for the repeatability and reproducibility were calculated.

### 2.9. Clinical Sample Detection

The RNA from 87 clinical samples was detected using the RF-RT-RAA assay in this study. It was also detected using a commercial PRRSV-2 real-time fluorescent RT-PCR detection kit (Beijing Anheal Laboratories Co., Ltd., Beijing, China) for comparison.

## 3. Results

### 3.1. Primers Screening and RF-RT-RAA Assay Establishment

**Figure 2:**
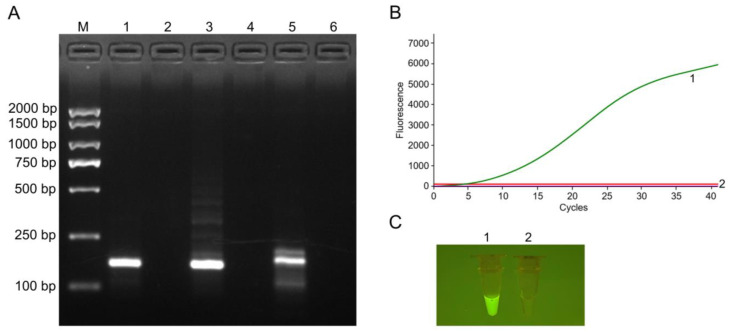
Primer screening and RF-RT-RAA assay establishment. (**A**) Agarose gel electrophoresis results of RT-RAA products using different primer pairs. M: DL2000 DNA marker; 1, 3, 5: Positive reactions using primer pairs of ORF6 F1/ORF6 R1, ORF6 F2/ORF6 R2, ORF6 F3/ORF6 R3, respectively; 2, 4, 6: Negative controls for primer pairs of ORF6 F1/ORF6 R1, ORF6 F2/ORF6 R2, ORF6 F3/ORF6 R3, respectively. (**B**) Amplification results of RF-RT-RAA through real-time fluorescence read-out. (**C**) Amplicons of RF-RT-RAA observed with the naked eye under blue light. The image was captured by a mobile phone camera. Curve or tube 1: Positive reaction using the primer pair ORF6 F1/ORF6 R1 and a probe; curve or tube 2: Negative control.

Three primer pairs were designed and used for the RT-RAA assay. Only ORF6 F1/ORF6 R1 generated a single and prominent band of the expected size on 2% agarose gel electrophoresis (Figure 2A). Consequently, ORF6 F1/ORF6 R1 was selected as the optimal primer pair, then a probe was designed based on it, and the primer/probe set was used for the RF-RT-RAA assay. Figure 2B displays that the positive reaction produced a significant fluorescence signal, while the negative control yielded no fluorescence signal. Additionally, the positive amplicon was visible with the naked eye under the portable blue light transilluminator (Figure 2C).

### 3.2. Specificity Test

**Figure 3:**
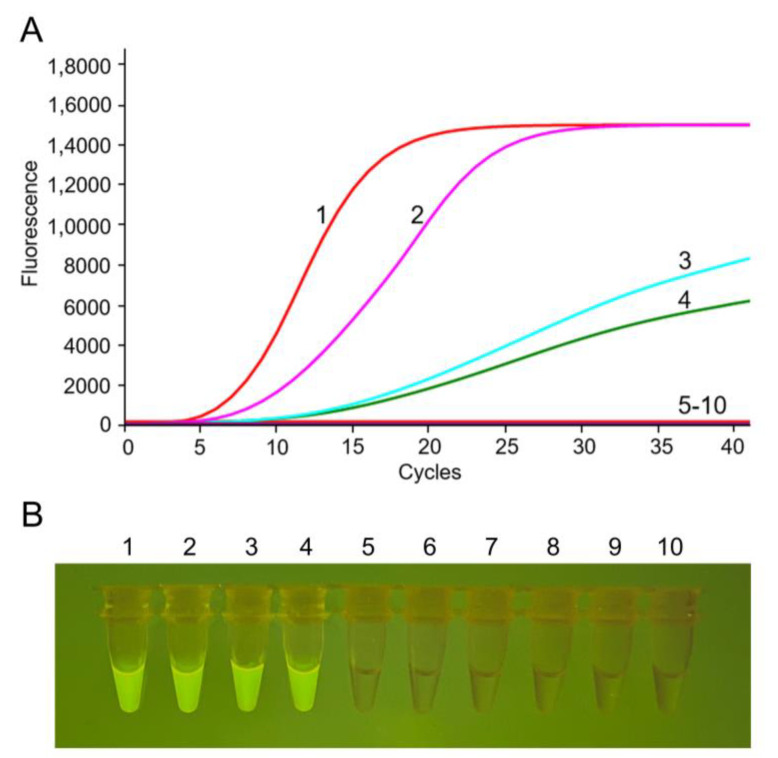
Specificity of the RF-RT-RAA assay for PRRSV-2. (**A**) Amplification results of RF-RT-RAA through real-time fluorescence read-out. (**B**) Amplicons of RF-RT-RAA observed with the naked eye under blue light. The image was captured by a mobile phone camera. Curves or tubes 1–4: RF-RT-RAA detection results of PRRSV-2 strains VR-2332 (RNA, 388 ng/μL), JS07 (RNA, 305 ng/μL), JXA1 (RNA, 206 ng/μL), and SH1705 (RNA, 178 ng/μL), respectively; curves or tubes 5–10: RF-RT-RAA detection results of PRV (DNA, 215 ng/μL), PEDV (RNA, 226 ng/μL), PCV2 (DNA, 264 ng/μL), TGEV (RNA, 314 ng/μL), PPV (DNA, 185 ng/μL), and negative control, respectively; PRV, porcine pseudorabies virus; PEDV, porcine epidemic diarrhea virus; PCV2, porcine circovirus type 2; TGEV, transmissible gastroenteritis virus; PPV, porcine parvovirus.

The nucleic acids extracted from PRRSV-2 and other swine viruses were used as the templates to test the specificity of RF-RT-RAA assay. Results demonstrated that the thermostatic fluorescence detector detected that all the PRRSV-2 strains were positive, while other viruses were negative (Figure 3A). The portable blue light transilluminator results were consistent with the form fluorescence detector read-out (Figure 3B). These results demonstrated that RF-RT-RAA assay had no cross-reaction with other common swine viruses, exhibiting a good specificity for PRRSV-2 detection.

### 3.3. Comparison of the Sensitivity of RF-RT-RAA and RT-PCR

**Figure 4:**
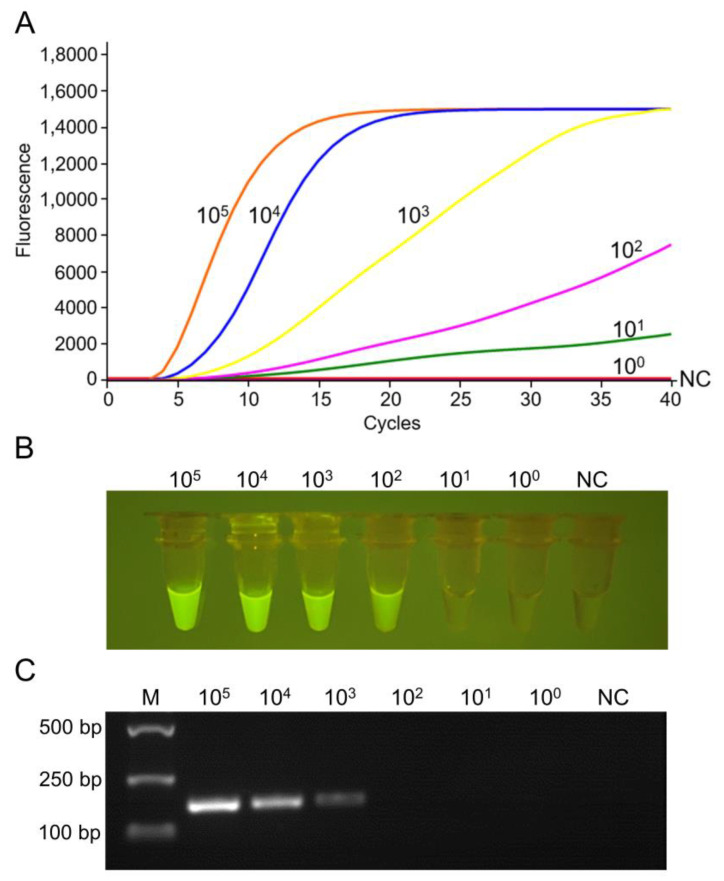
Sensitivity of RF-RT-RAA and RT-PCR for PRRSV-2 detection. (**A**) Sensitivity test of RF-RT-RAA through real-time fluorescence read-out. (**B**) Sensitivity test of RF-RT-RAA through visual observation. The image was captured by a mobile phone camera. (**C**) Sensitivity test of RT-PCR. NC, negative control.

Serial dilutions of standard plasmid were used as templates for detecting RF-RT-RAA and RT-PCR. Results revealed that the RF-RT-RAA lowest limit was 10^1^ copies/μL detected through real-time fluorescence read-out (Figure 4A). When the amplicon was visually observed by a portable blue light transilluminator, it was 10^2^ copies/μL (Figure 4B). The reaction conditions of RT-PCR were optimized (Figure S1), and its lowest detection limit was 10^3^ copies/μL (Figure 4C), indicating that the RF-RT-RAA assay was more sensitive than RT-PCR. Additionally, the lowest detection limit of RF-RT-RAA for viral RNA was 10^0.5^ TCID_50_/mL through real-time fluorescence read-out, and 10^1.5^ TCID_50_/mL via visual observation under blue light (Figure S2).

### 3.4. Repeatability and Reproducibility Tests

Table 2 displays that the intra-assay repeatability CV was 3.66–5.64%, and the inter-assay reproducibility CV was 5.47–6.51%. All CV values were less than 10%, indicating that RF-RT-RAA assay had good repeatability and reproducibility.

### 3.5. Clinical Sample Detection

Eighty-seven clinical samples were tested for PRRSV-2 using the established RF-RT-RAA assay, and a commercial PRRSV-2 RT-qPCR kit was used as a standard for comparison. Table 3 shows that the positive/negative samples of RF-RT-RAA (real-time fluorescence read-out), RF-RT-RAA (visually observed), and RT-qPCR were 46/41, 44/43, and 46/41, respectively. The RF-RT-RAA (real-time fluorescence read-out) and RT-qPCR detection results were completely consistent. Additionally, all 44 positive samples tested by RF-RT-RAA (visually observed) were also identified positively by RT-qPCR. However, out of 43 negative samples tested by RF-RT-RAA (visually observed), 41 displayed negative results by RT-qPCR, generating a coincidence rate of 97.7% between the two methods. These results indicated that RF-RT-RAA assay for PRRSV detection could be applied in clinical practice.

## 4. Discussion

Despite numerous efforts, PRRS remains one of the most important diseases affecting the global swine industry [22]. Early and accurate detection is important for PRRSV prevention and control. Many laboratories have used molecular biology methods such as RT-PCR and RT-qPCR to detect PRRSV. RT-PCR and RT-qPCR have good specificity and sensitivity. Still, they are limited by expensive thermal cyclers and time-consuming procedures, restricting their applications in under-equipped laboratories or fields. To overcome these disadvantages, several methods have been developed for PRRSV detection based on isothermal nucleic acid amplification, mainly including reverse transcription loop-mediated isothermal amplification (RT-LAMP) and reverse transcription recombinase polymerase amplification (RT-RPA) [14,23,24]. To the best of our knowledge, this is the first report on PRRSV detection by RF-RT-RAA.

Previously, an RT-LAMP assay for PRRSV-2 detection was established [14]. The reaction procedure of RT-LAMP was incubated at 63.5 °C for 60 min in a heating block, then at 80 °C for 2 min to terminate the reaction. For comparison, our study could conduct RF-RT-RAA assay at a lower temperature (42 °C) for a shorter time (20 min). Moreover, RT-LAMP assay requires six primers which are challenging to design, while the RF-RT-RAA reaction system only needs two primers and a probe. In addition to RT-LAMP, RT-RPA-based methods were developed for PRRSV-2 detection, including real-time RT-RPA [24] and RT-RPA combined with a lateral flow dipstick (RT-RPA-LFD) [25]. Real-time RT-RPA and RT-RPA-LFD assays for PRRSV detection could complete the amplification at a constant temperature of 40–42 °C within 20–30 min, similar to the RF-RT-RAA method developed in this study. RPA and RAA share a similar principle and process but use different recombinases. The RPA recombinase is derived from a T4 bacteriophage, while the RAA recombinase is taken from bacteria or a fungus, which has a broader range of sources and lower costs [26]. In addition, the RAA recombinase can bind to primers closely even at room temperature [17]. Notably, RT-RPA-LFD amplification products could be visually observed with the naked eye using an LFD, providing a simple alternative for PRRSV detection. However, the lid must be opened during the LFD detection step, which may induce aerosol pollution from amplicons. In our study, the RF-RT-RAA assay tubes were closed after amplification, thus preventing cross-contamination.

In previous studies, the real-time fluorescence-based RAA/RT-RAA assays were usually performed on real-time PCR instruments in professional laboratories [21,27]. In order to make the RF-RT-RAA method more convenient for on-site detection of PRRSV, we chose a portable thermostatic fluorescence detector to conduct the assay, and the reaction could be completed at 42 °C in 20 min with a high sensitivity of 10^1^ copies/μL. The sensitivity was consistent with the RT-qPCR method reported in the literature [28] and 100 times higher than RT-PCR. Additionally, the reaction condition and sensitivity were similar to other RAA/RT-RAA studies [19,27], indicating that RAA/RT-RAA is expected to be a general technique for pathogen detection. Furthermore, to better apply the RF-RT-RAA assay for field use, a portable blue light transilluminator was employed for visual detection. We were able to directly observe the amplification products of RF-RT-RAA under blue light. Although the lowest detection limit of RF-RT-RAA via visual observation was 10^2^ copies/μL, it was still 10 times more sensitive than RT-PCR. Notably, this simple way does not require a fluorescence detector, and the reaction can be conducted with a water bath, which is also very suitable for resource-poor laboratories.

Additionally, the developed RF-RT-RAA assay yielded a strong specificity that no cross-reaction was identified with the common swine viruses such as PRV, PEDV, PCV2, TGEV, and PPV. In addition, the technique exhibited good repeatability and reproductively with the CV values less than 10%. Accordingly, the RF-RT-RAA assay appears to be a reliable diagnostic tool for PRRSV-2 detection. Furthermore, a commercial PRRSV-2 RT-qPCR was used as a comparison to validate RF-RT-RAA performance for clinical sample detection. The coincidence rate was 100% between RF-RT-RAA (real-time fluorescence read-out) and RT-qPCR, and 97.7% between RF-RT-RAA (visually observed) and RT-qPCR, indicating that RF-RT-RAA had great potential for clinical practice. However, considering that the RF-RT-RAA assay was performed in a one-step procedure for reverse transcriptase (RT) and RAA amplification, it is important to ensure that the RT step is successfully completed in clinical detection. In the present study, a template of total RNA extracted from a PRRSV-positive clinical sample (detected by RT-qPCR) was used as a positive control in each RF-RT-RAA assay. In future work, we will further set an internal control (such as a porcine housekeeping gene, GAPDH) in RF-RT-RAA assay for clinical detection.

## 5. Conclusions

A rapid and visual method for PRRSV-2 detection by RF-RT-RAA was developed. RF-RT-RAA assay could be performed at 42 °C for 20 min with good specificity and sensitivity, and its results could be detected via a real-time fluorescence detector or a blue light transilluminator. The novel method provides a new approach to the clinical diagnosis of PRRSV-2 infection, especially in resource-poor laboratories or fields.

## Figures and Tables

**Table 1:** Primers and probe used in this work.

Primer/ Probe	Sequence (5′-3′)	Position	Product Size (bp)
ORF6 F1	CGCAAGTACATTCTGGCCCCTGCCCACCAC	316–345	161
ORF6 R1	CTGCCACCCAACACGAGGCCATTCAACCCG	447–476	
ORF6 F2	GCCGCAAGTACATTCTGGCCCCTGCCCACCA	314–344	165
ORF6 R2	TTCTGCCACCCAACACGAGGCCATTCAACC	449–478	
ORF6 F3	TTGTGCTTGCTAGGCCGCAAGTACATTCTG	301–330	174
ORF6 R3	GCCACCCAACACGAGGCCATTCAACCCGGG	445–474	
Probe	CGATTGCGGCAAATGATAACCACGCATTTG /i6FAMdT//THF/G/iBHQ1dT/CCGGCGTCCCGGCT (C3-Spacer)	371–418	

**Table 2:** Repeatability and reproducibility of RF-RT-RAA for PRRSV-2 detection.

Templates (Copies/μL)	Repeatability		Reproducibility	
	Ct (Mean ± SD)	CV	Ct (Mean ± SD)	CV
10^3^	4.43 ± 0.25	5.64%	4.30 ± 0.26	6.05%
10^2^	5.73 ± 0.21	3.66%	5.67 ± 0.31	5.47%
10^1^	8.13 ± 0.45	5.54%	7.83 ± 0.51	6.51%

**Table 3:** Clinical performance of RF-RT-RAA for PRRSV-2 detection compared with RT-qPCR.

Methods		RT-qPCR			Coincidence Rate
		Positive	Negative	Total	
RF-RT-RAA (real-time fluorescence read-out)	Positive	46	0	46	100%
	Negative	0	41	41	
	Total	46	41	87	
RF-RT-RAA (visually observed)	Positive	44	0	44	97.7%
	Negative	2	41	43	
	Total	46	41	87	

## Data Availability

The data presented in this study are available in the article or Supplementary Materials.

## Supplementary Materials

The following supporting information can be downloaded at: https://www.mdpi.com/article/10.3390/v14112526/s1, Figure S1: Optimization of reaction conditions for PRRSV detection of RT-PCR; Figure S2: Sensitivity of RF-RT-RAA for PRRSV-2 detection using viral RNA templates.

## References

[1] Kappes M.A., Faaberg K.S. (2015). PRRSV structure, replication and recombination: Origin of phenotype and genotype diversity. Virology.

[2] Lunney J.K., Benfield D.A., Rowland R.R. (2010). Porcine reproductive and respiratory syndrome virus: An update on an emerging and re-emerging viral disease of swine. Virus Res..

[3] Wensvoort G., Terpstra C., Pol J.M., Ter Laak E.A., Bloemraad M., de Kluyver E.P., Kragten C., van Buiten L., den Besten A., Wagenaar F. (1991). Mystery swine disease in The Netherlands: The isolation of Lelystad virus. Vet. Q..

[4] Nelsen C.J., Murtaugh M.P., Faaberg K.S. (1999). Porcine reproductive and respiratory syndrome virus comparison: Divergent evolution on two continents. J. Virol..

[5] Jiang Y., Li G., Yu L., Li L., Zhang Y., Zhou Y., Tong W., Liu C., Gao F., Tong G. (2020). Genetic Diversity of Porcine Reproductive and Respiratory Syndrome Virus (PRRSV) From 1996 to 2017 in China. Front. Microbiol..

[6] Tian K. (2017). NADC30-Like Porcine Reproductive and Respiratory Syndrome in China. Open Virol. J..

[7] Zhang H.L., Zhang W.L., Xiang L.R., Leng C.L., Tian Z.J., Tang Y.D., Cai X.H. (2018). Emergence of novel porcine reproductive and respiratory syndrome viruses (ORF5 RFLP 1-7-4 viruses) in China. Vet. Microbiol..

[8] Xu H., Song S., Zhao J., Leng C., Fu J., Li C., Tang Y.D., Xiang L., Peng J., Wang Q. (2020). A potential endemic strain in China: NADC34-like porcine reproductive and respiratory syndrome virus. Transbound. Emerg. Dis..

[9] Xia W., Wu Z., Guo C., Zhu S., Zhang X., Xia X., Sun H. (2018). Recombinant adenovirus-delivered soluble CD163 and sialoadhesin receptors protected pigs from porcine reproductive and respiratory syndrome virus infection. Vet. Microbiol..

[10] Xiao Y.H., Wang T.T., Zhao Q., Wang C.B., Lv J.H., Nie L., Gao J.M., Ma X.C., Hsu W.H., Zhou E.M. (2014). Development of indirect ELISAs for differential serodiagnosis of classical and highly pathogenic porcine reproductive and respiratory syndrome virus. Transbound. Emerg. Dis..

[11] Peng Z., Zhao T., Liang W., Song W., Gao Z., Tang X., Chen H., Wu B. (2017). RT-PCR detection of porcine reproductive and respiratory syndrome virus based on the ORF5 gene in mainland China, 2012–2015. Acta Virol..

[12] Xiao S., Chen Y., Wang L., Gao J., Mo D., He Z., Liu X. (2014). Simultaneous detection and differentiation of highly virulent and classical Chinese-type isolation of PRRSV by real-time RT-PCR. J. Immunol. Res..

[13] Zhang H.L., Tang Y.D., Liu C.X., Xiang L.R., Zhang W.L., Leng C.L., Wang Q., An T.Q., Peng J.M., Tian Z.J. (2018). Adaptions of field PRRSVs in Marc-145 cells were determined by variations in the minor envelope proteins GP2a-GP3. Vet. Microbiol..

[14] Zhang L., Liu Y.B., Chen L., Wang J.H., Ning Y.B. (2011). Rapid and sensitive detection of PRRSV by a reverse transcription-loop-mediated isothermal amplification assay. Virol. Sin..

[15] Wang W., Wang C., Zhang Z., Zhang P., Zhai X., Li X., Zhang T. (2021). Recombinase-aided amplification-lateral flow dipstick assay-a specific and sensitive method for visual detection of avian infectious laryngotracheitis virus. Poult. Sci..

[16] Xia W., Chen K., Liu W., Yin Y., Yao Q., Ban Y., Pu Y., Zhan X., Bian H., Yu S. (2022). Rapid and visual detection of Mycoplasma synoviae by recombinase-aided amplification assay combined with a lateral flow dipstick. Poult. Sci..

[17] Fan X., Li L., Zhao Y., Liu Y., Liu C., Wang Q., Dong Y., Wang S., Chi T., Song F. (2020). Clinical Validation of Two Recombinase-Based Isothermal Amplification Assays (RPA/RAA) for the Rapid Detection of African Swine Fever Virus. Front. Microbiol..

[18] Li X., Wang C., Zhang Z., Wang C., Wang W., Zhao Z., Li J., Shang Z., Lv J., Zhang T. (2022). Fast detection of duck circovirus by real-time fluorescence-based recombinase-aided amplification. Poult. Sci..

[19] Wu T., Ge Y., Zhao K., Zhu X., Chen Y., Wu B., Zhu F., Zhu B., Cui L. (2020). A reverse-transcription recombinase-aided amplification assay for the rapid detection of N gene of severe acute respiratory syndrome coronavirus 2(SARS-CoV-2). Virology.

[20] Wu X., Liu Y., Gao L., Yan Z., Zhao Q., Chen F., Xie Q., Zhang X. (2022). Development and Application of a Reverse-Transcription Recombinase-Aided Amplification Assay for Porcine Epidemic Diarrhea Virus. Viruses.

[21] Tu F., Yang X., Xu S., Chen D., Zhou L., Ge X., Han J., Zhang Y., Guo X., Yang H. (2021). Development of a fluorescent probe-based real-time reverse transcription recombinase-aided amplification assay for the rapid detection of classical swine fever virus. Transbound. Emerg. Dis..

[22] Ruedas-Torres I., Rodriguez-Gomez I.M., Sanchez-Carvajal J.M., Larenas-Munoz F., Pallares F.J., Carrasco L., Gomez-Laguna J. (2021). The jigsaw of PRRSV virulence. Vet. Microbiol..

[23] Gao M., Cui J., Ren Y., Suo S., Li G., Sun X., Su D., Opriessnig T., Ren X. (2012). Development and evaluation of a novel reverse transcription loop-mediated isothermal amplification (RT-LAMP) assay for detection of type II porcine reproductive and respiratory syndrome virus. J. Virol. Methods.

[24] Wang J.C., Yuan W.Z., Han Q.A., Wang J.F., Liu L.B. (2017). Reverse transcription recombinase polymerase amplification assay for the rapid detection of type 2 porcine reproductive and respiratory syndrome virus. J. Virol. Methods.

[25] Tian X.X., Wang T., Cui X.Y., Huang X.Y., Sun Y., Xia D.S., Yang Y.B., Cai X.H., An T.Q. (2022). Rapid visual detection of porcine reproductive and respiratory syndrome virus via recombinase polymerase amplification combined with a lateral flow dipstick. Arch. Virol..

[26] Chen W., Fan J., Li Z., Zhang Y., Qin Y., Wu K., Li X., Li Y., Fan S., Zhao M. (2021). Development of Recombinase Aided Amplification Combined With Disposable Nucleic Acid Test Strip for Rapid Detection of Porcine Circovirus Type 2. Front. Vet. Sci..

[27] Wang W., Wang C., Zhang Z., Zhang P., Yao S., Liu J., Zhai X., Zhang T. (2020). Research Note: Rapid detection of avian infectious laryngotracheitis virus with real-time fluorescence-based recombinase-aided amplification. Poult. Sci..

[28] Qiu W., Meng K., Liu Y., Zhang Y., Wang Z., Chen Z., Yang J., Sun W., Guo L., Ren S. (2019). Simultaneous detection of classical PRRSV, highly pathogenic PRRSV and NADC30-like PRRSV by TaqMan probe real-time PCR. J. Virol. Methods.

