# Supplementary material for: Rapid and Visual Detection of Type 2 Porcine Reproductive and Respiratory Syndrome Virus by Real-Time Fluorescence-Based Reverse Transcription Recombinase-Aided Amplification
Source: Viruses. 2022 Nov 15;14(11):2526. doi: 10.3390/v14112526 (PMC9699348; doi:10.3390/v14112526)
Supplement: Supplementary file 1 [file viruses-14-02526-s001.zip › viruses-2002831-supplementary.pdf]

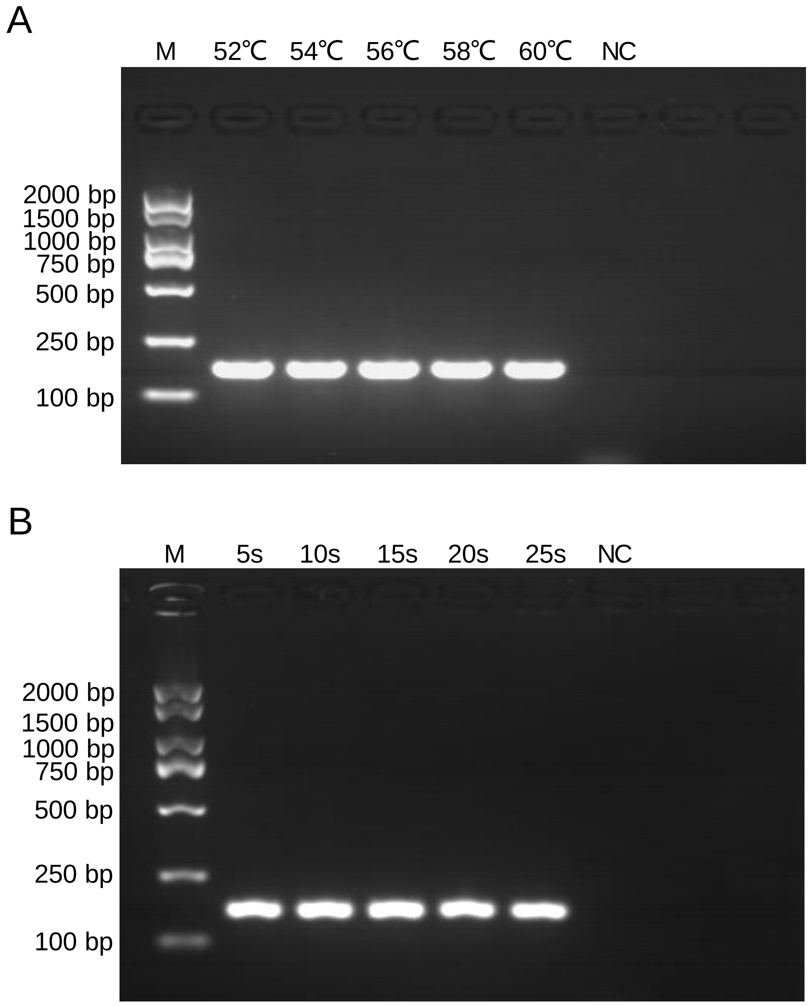

Figure S1. Optimization of reaction conditions for PRRSV detection of RT-PCR. **(A)** Agarose gel electrophoresis results of RT-PCR under different annealing temperatures. The bands were similar to each other, and 56 °C were used in the final program. **(B)** Agarose gel electrophoresis results of RT-PCR using different extension times. The band from an extension time of 15 s was relatively brighter, so the final extension time of RT-PCR program was set as 15 s. NC, negative control. The cDNA of PRRSV JXA1 was used as a template for each PCR reaction.

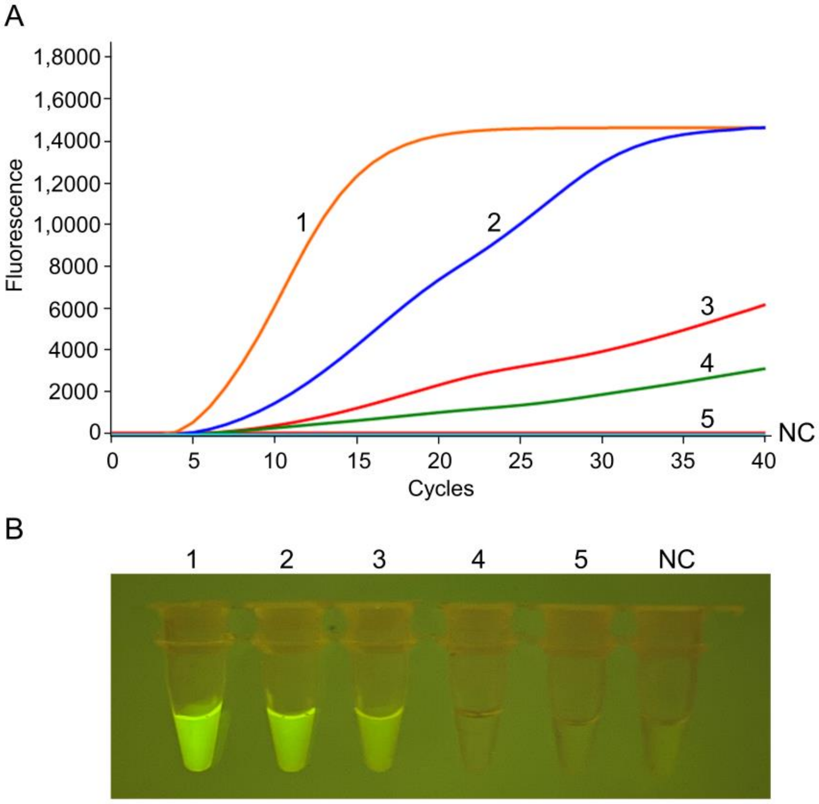

Figure S2. Sensitivity of RF-RT-RAA for PRRSV-2 detection using viral RNA templates. **(A)** Sensitivity test of RF-RT-RAA through real-time fluorescence read-out. **(B)** Sensitivity test of RF-RT-RAA through visual observation. The image was captured by a mobile phone camera. Curves or tubes 1-5: RF-RT-RAA detection results with viral RNA templates of $10^{3.5}$–$10^{-0.5}$ $TCID_{50}$/mL, respectively. NC, negative control.
